# Protective effect of Sheng-Mai Yin, a traditional Chinese preparation, against doxorubicin-induced cardiac toxicity in rats

**DOI:** 10.1186/s12906-016-1037-9

**Published:** 2016-02-11

**Authors:** Shaojun Ma, Xiaojiang Li, Liang Dong, Jinli Zhu, He Zhang, Yingjie Jia

**Affiliations:** Department of Oncology, Tianjin Nankai Hospital, 122 San Wei Lu, Nan Kai District, Tianjin, 300100 China; Department of Oncology, The First Affiliated Hospital to Tianjin University of Traditional Chinese Medicine, 314 An Shan Xi Dao, Nan Kai District, Tianjin, 300193 China

**Keywords:** Doxorubicin, Myocardial fibrosis, Sheng-Mai Yin

## Abstract

**Background:**

Sheng-Mai Yin (SMY), a modern Chinese formula based on Traditional Chinese Medicine theory, has been used to treat cardiovascular diseases in Eastern Asia. Our study focuses on the cardioprotection of SMY against doxorubicin (DOX)-induced cardiac toxicity in vivo.

**Methods:**

Rats were injected with DOX (2.5 mg/kg) in six injections over a 2-week period. SMY was administrated intragastrically at the dose of 8.35, 16.7 and 33.4 g/kg, or 16.7 g/kg only twice a day concurrently with DOX for the 2-weeks. A series of assays were performed to detect the effects of SMY on: (i) heart weight index (HWI) and left ventricular mass index (LVMI); (ii) cardiac function; (iii) heart tissue morphology; (iv) the contents of carboxy terminal propeptide of procollagen typeI (PICP), amino terminal propeptide of procollagen type III (PШNP), transforming growth factor-β1 (TGF-β1), B-type natriuretic peptide (BNP), monocyte chemoattractant protein-1 (MCP-1), interferon gamma (INF-γ) and interleukin 6 (IL-6) by ELISA; (v) the mRNA levels of TGF-β1 and toll-like receptor-2 (TLR2); and (vi) protein level of TGF-β1.

**Results:**

Rats treated with SMY displayed the reductions of BNP and CK-MB increased by DOX in a dose-dependent manner. Moderate dose of SMY exhibited the correction for the increased HWI, LVMI, and the injured cardiac function, as well as the collagen accumulation. In addition, cardioprotection of SMY against DOX-induced cardiac toxicity was demonstrated by the reduction of myocardial fibrosis, characterized by the suppression of PICP, PШNP and TGF-β1, as well as the anti-inflammation and the regulation for cardiac immune microenvironment, characterized by the inhibition of TLR2, MCP-1, INF-γ and IL-6.

**Conclusions:**

SMY may protect heart function through the restriction of myocardial fibrosis induced by DOX, which suggests the potentially therapeutic effect of SMY on DOX-induced cardiomyopathy.

## Background

Doxorubicin (DOX), a broad spectrum anthracycline antineoplastic, is widely used for the treatment of various malignancies such as acute leukemia, lymphoma and breast cancer [1, 2]. Unfortunately, clinical applicationsof this drug are restricted due to the severe, dose-dependent, acute cardiotoxicity that may involve various signaling pathways including free radical generation, peroxynitrite formation, calcium overloading, mitochondrial dysfunction, alteration in beta-adrenergic receptor signaling, and activation of matrix metalloproteinase [3, 4] which will inevitably lead to congestive heart failure [2, 5]. Moreover, some studies have found that patients receiving DOX therapy had an increased risk of myocardial infarction (MI) and this risk persisted up to 25 years after DOX treatment [6]. Over the past decade, adjuvant therapies have been proposed to decrease the cardiotoxic effects of DOX. For instance, the use of antioxidants such as probucol, taurine and fenofibrate has been shown to suppress DOX-induced oxidative stress and cardiac myocyte apoptosis [7–9]. Despite various therapeutic interventions adopted to protect the heart against DOX-induced cardiotoxicity, the deterioration in cardiac functions is often accompanied by high mortality rates [10]. Hence, further therapeutic agent development especially the pharmacodynamic constituents from natural herb in complementary and alternative medicine (CAM) have been regarded as the focus in the amelioration of DOX-induced cardiac damage.

Traditional Chinese medicine (TCM) has a 3000-year-old history that includes unique theories for aetiology and systems of diagnosis and treatment. Nowadays, it is widely accepted that multiple ingredients counteract chemotherapy-induced gastrointestinal toxicity and kansui-induced hepatocyte cytotoxicity [11, 12]. Sheng-Mai Yin (SMY), a traditional Chinese formula widely used for coronary heart disease treatment with “qi–yin” deficiency, consists of three herbal materials of *Radix ginseng* (invigorates Qi and dispels stagnation), *Radix ophiopogonis* (nourishes Yin and grows succus) and *Fructus schisandrae* (invigorates Qi) [13]. A series of animal studies have reported that Shengmai preparation could improve the glutathione peroxidase (GSH-Px) activities, superoxide dismutase (SOD) activity [14], Ca^2+^-ATP enzyme activity and myocardial ultrastructure [15] of DOX-injured rats myocardial tissue. In addition, anti-apoptosis effect of Shengmai injection was also observed in DOX-induced rats, characterized by the reduction of Bax protein and the increase of Bcl-2 protein [16]. Consequently, it seemed that Shengmai treatment had an effect in DOX-induced cardiac toxicity model. Although there is a lot of research regarding SMY’s cardioprotecion, few of them involved in the myocardial fibrosis induced by DOX. Therefore, this study investigated protective effects of SMY on DOX-induced myocardial fibrosis. The rat models induced by DOX were employed and many pharmacological indicators including electrocardiogram, cardiac function and myocardial fibrosis analysis were observed.

## Methods

### Animals

The study protocol was approved by the Institutional Animal Care and Use Committee of Tianjin University of Traditional Chinese Medicine and in accordance with the principles outlined in the NIH Guide for the Care and Use of Laboratory Animals. Thirty adult Sprague– Dawley male rats (SPF grade) weighing 235 ± 20 g, 7- to 8-week-old, were kept in standard cages at 25 ± 1 °C under a 12-h light/dark cycle and fed a rodent standard diet with free access to water. According to the mean and standard deviation of PICP in preliminary experiment, we calculated the sample size as 24 rats with software SAS9.2 (α = 0.05,β = 0.1, power = 0.9). Considering the possible animal deaths in the 2-weeks, we involved totally 30 rats in the experiment. The rats were numbered and randomized into three groups according to the random number generated by rand function of EXCEL: control group (*n* = 10), DOX group (*n* = 10), and DOX + SMY group (*n* = 10). 10 rats in DOX group were administered DOX and 10 rats in DOX + SMY group were administered DOX + SMY.

### Sheng-Mai Yin (SMY) preparation

The SMY was composed by *Radix ginseng, Radix ophiopogonis* and *Fructus schisandrae* at the ration of 1:2:1. To keep the consistency of the herbal chemical ingredients, all of the herbal components were originally obtained from the standard native sources as stated above with GAP grade. All of these herbal materials (Heyanling Chinese Herbal Medicine Co., Ltd., Beijing, China) were the same batch during the whole experimental processand were identified by School of Pharmacy, Tianjin University of Traditional Chinese Medicine. A voucher specimen of each species was also deposited at Tianjin University of Traditional Chinese Medicine. To prepare the SMY [17], the mixture of the three herbs were pulverized to coarse powder, macerated in 65 % (vol/vol, in H_2_O) ethanol (11.5 mL/g) for 24 h, and then extracted by percolation. The extracts were concentrated, followed by removing undissolved particulates by filtration. The mixture was adjusted to pH 6. The liquid of SMY was sterilized by 0.22 μm filter before the administration. Using these procedures, the total concentration of the SMY was 0.375 g/mL (crude drug content) based on data from processing and stability studies. The SMY was stored at 4 °C for use.

### Drugs and animal treatment

Rats were treated with DOX (Sigma, St. Louis, MO, USA) as previously reported with modification. In brief, rats were injected i.p. with DOX (2.5 mg/kg) dissolved in normal saline every other day (Monday, Wednesday, and Friday) over a 2 week period (total six injections) to produce a total cumulative dose of 15 mg/kg body weight [18]. In clinical, the adult dose of Shengmai Yin was 2.0875 g/kg and the equivalent in rats was 16.7 g/kg which was used as moderate dose. In treatment group, rats were given SMY concurrently with DOX at the dose of 8.35, 16.7 and 33.4 g/kg as the low, moderate and high doses respectively (moderate dose was converted according to the clinical adult dosage) twice a day during the 2-week period via intragastric administration. Therats in high-dosage and low-dosage group received SMY at a dose 2 fold moderate and half a moderate, respectively (33.4 g/kg, 8.35 g/kg). Control animals received the vehicle (saline) only.

### Body weight, heart weight index (HWI) and left ventricular mass index (LVMI) calculation

The body weight of rat was recorded everyday. All the rats had echocardiographic assessment and were sacrificed the day after the last SYM administration. Heart tissues were excised, rinsed with cold PBS solution, and the left ventricle was separated from the atria, aorta and adipose tissue. The left ventricle weight (LVW, mg) and heart weight (HW, mg) were weighed, and then left ventricular weight index (LVMI, mg/g) and heart weight index (HWI, mg/g) were calculated by the ratios of the LVW to the body weight and the HW to the body weight, respectively.

### Electrocardiography and the echocardiography

The ECG was monitored at the end of experiment by Biopac MP150 data acquisition system. Cardiac function was determined by transthoracic echocardiography as previously described [19]. The rats were anesthetized with 1 % sodium pentobarbital and were fixed on the board. The hairs of rats were removed in the ventral chest area and front area. There is the echocardiography probe which emits and receives ultrasound in the range of 5–12 MHz. The LVEDD and LVESD were measured. The EF was calculated using the formula as follows: EF = [(LVEDD^3^ − LVEDS^3^)/LVEDD^3^] × 100 % [20]. For each rat, two measurements were performed: 1 day before the onset of experiment and the end of experiment.

### Morphological examination

The histopathological evaluations including hematoxylin-eosin staining (H&E) and Masson staining in each group were conducted by standard histological techniques. The heart tissues were fixed in 10 % buffered formalin and embedded in paraffin. The 5 μm sections were stained with H&E. As for the Masson staining, sections were incubated in Bouins Fixative for 45 min, washed and stained with weigert iron hematoxylin, anylin blue, and finally mounted with cytoseal-60 and cover slip. Each slide was analyzed under a brightfield microscope with the fibrotic areas stained in blue and the healthy in red. All photos were analyzed with the image-proplus 6.3 analyzing software (Media Cybernetics, Bethesda, MD, USA) by computer.

### Enzyme-linked immunosorbent assay (ELISA)

Serum or plasma was prepared from peripheral blood. Heart tissues were removed, homogenated with ice-cold PBS, centrifuged at 12,000 rpms for 20 min and separated the supernatant. The levels of procollagen type 1 (PICP), amino terminal propeptide of procollagen type III (PIIINP) and B-type natriuretic peptide (BNP) levels in serum, as well as transforming growth factor-β1 (TGF-β1), monocyte chemoattractant protein-1 (MCP-1), interferon gamma (INF-γ) and interleukin 6 (IL-6) in the heart tissue were measured by ELISA according to the manufacture instructions and calculated according to the standard curve. Creatine kinase MB isoenzyme (CK-MB) level in the plasma was determined by spectrophotometry.

### mRNAs detection

TGF-β1 mRNA expression in the heart tissue was observed by Reverse transcription (RT) polymerase chain reaction (PCR) as described [21]. In short, total RNA from heart tissue was extracted using TRIZOL reagent (Invitrogen). Two μL of total RNA were used for reverse transcription to obtain cDNA by a cDNA synthesis kit (Fermentas). Each cDNA was used successively to amplify TGF-β1 and glyceraldehyde 3-phosphate dehydrogenase (GAPDH) (loaded as a control). Amplification was performed with a reaction protocol of AmpliTaqDNA polymerase (Fermentas) at 94 °C for 3 min followed by 30 cycles at 94 °C (40 s), 60 °C (30 s), and 72 °C (45 s) and 72 °C (10 min). The bands of PCR products separated by agarose gel electrophoresis (BioWest) were analyzed via the IS-1000 analyzer system.cDNA was also subjected to real-time quantitative PCR with toll-like receptor-2 (TLR2) primers and Power SYBR Green PCR Master Mix (Applied Biosystems, Foster City, CA, USA) using the ABI 7000 sequence detection system (Applied Biosystems). The data of TLR2 was analyzed using the ABI 7000 system SDS software and determined by the 2^−ΔΔCt^ method. The sequences of primers were shown as follows: TGF-β1 (5′- GGCGGTGCTCGCTTTGT-3′/5′- GCCCTGTATTCCGTCTCCTT −3′), fragment length was 424 bp. TLR2 (5′- ATGCTGCAAGCTCTTTGGCTCTTC-3′/5′- AGTCACCAfGGCCAATGTAGGTG −3′), fragment length was 241 bp. β-actin (5′- TGTGCTATGTTGCCCTAGACT −3′/5′- ACGTACTCCTGGTTGCTGAT −3′), fragment length was 493 bp. GAPDH (5′- AGAAGGCTGGGGCTCATTTG-3′/5′- AGGGGCCATCCACAGTCTTC-3′), fragment length was 257 bp.

### Western blots

TGF-β1 expression in the heart tissue was conducted by western blots according to standard protocols. Briefly, heart tissues were rinsed with ice-cold PBS and scraped in lysis buffer. The insoluble material was removed by centrifugation at 12,000 rpms for 20 min. Fifty micrograms protein was processed by SDS-PAGE separation in 12 % gel, followed by the transfer to a 0.45 μm nitrocellulose membrane. Tris–HCl buffered saline (TBS) containing 5 % nonfat dry milk was used to block the non-specific binding sites. The membrane was then incubated with primary antibodies (1:2000 dilution) against TGF-β1, or against β-actin (1:5000 dilution) as controls, followed by incubation with the corresponding horseradish peroxidase (HRP)-conjugated secondary antibodies (1:10,000 dilution). Immunoreactive proteins were detected by enhanced chemiluminescence according to the instructions of the manufacturer (Pierce, Rockford, IL).

### Statistical analysis

The experimental data were represented as mean ± standard deviation (SD). One way analysis of variance (ANOVA) was used to determine statistically significant differences among the groups. A *P*-value of <0.05 was considered to be statistically significant.

## Results

### Effects of SMY with different doses on BNP and CK-MB

**Fig. 1:**
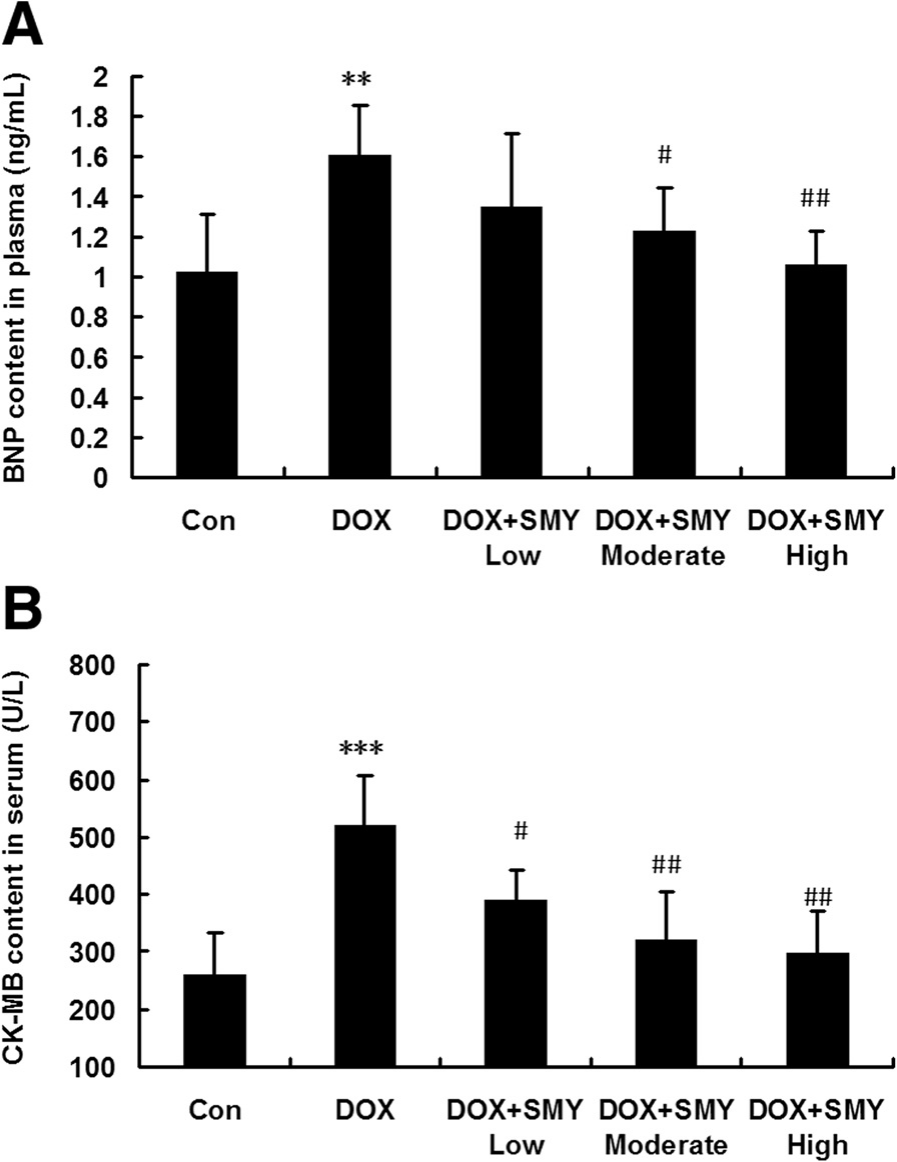
**a** Bar graphs showed the changes of BNP content determined via ELISA assay. SMY of both moderate dose and high dose suppressed the BNP content, with a significant reduction as compared to group DOX. **b** Bar graphs show the changes of CK-MB in serum. The CK-MB level was significantly inhibited by the SMY in a dose-dependent manner. Eight to ten rats in each group were analyzed in this experiment and all data were represented as mean ± standard deviation. ** *P* < 0.01 versus the group control, *** *P* < 0.001 versus the group control; #*P* < 0.05 versus the group DOX; ##*P* < 0.01 versus the group DOX

The gray scale image in Fig. 1 displayed the obvious increases of BNP and CK-MB in DOX group which suggests cardiac injury. However, treatment with SMY with low, moderate and high dose significantly inhibited the level of BNP in a dos -dependent manner (Fig. 1a, *P* < 0.05 or *P* < 0.01), with the reduction of 16.29 %, 23.28 % and 33.97 % respectively. Meanwhile, SMY also suppressed CK-MB content in a dose dependent manner (Fig. 1b, *P* < 0.05 or *P* < 0.01). Based on the above results, we chose the moderate dose of 16.7 g/kg in the following experiments.

### Effects of SMY on cardiac weight indexes

**Fig. 2:**
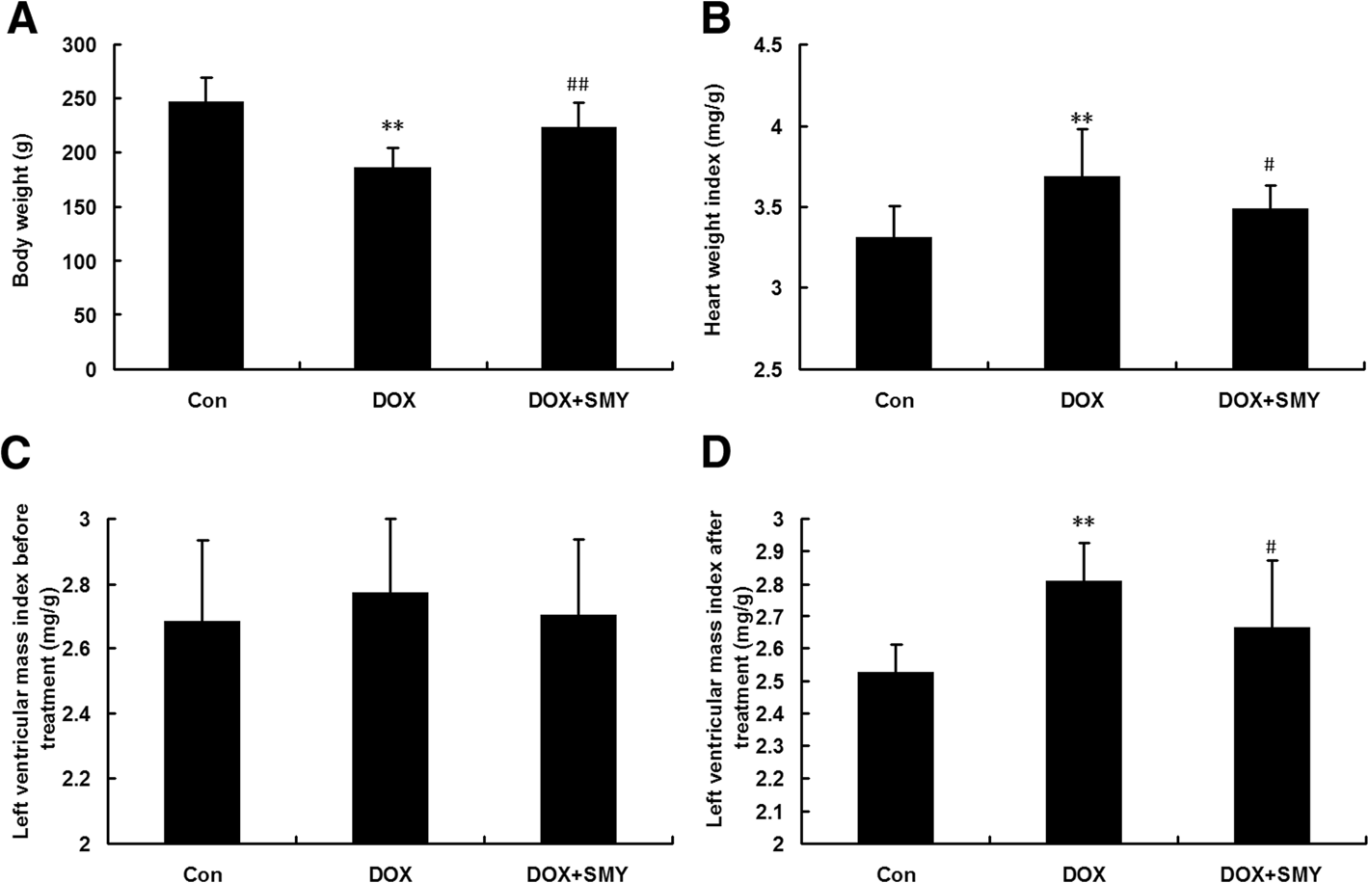
Effects of SMY on body weight (**a**), heart weight index (**b**) and left ventricular mass index (**d**) in rats with DOX-induced cardiomyopathy. **c** Baseline of left ventricular mass index before treatment. Ten rats in each group were analyzed in this experiment and all data were represented as mean ± standard deviation. ** *P* < 0.01 versus the group control; #*P* < 0.05 versus the group DOX; ##*P* < 0.01 versus the group DOX

The effects of SMY on HWI (HW/body weight) and LVMI (LVW/ body weight) were shown in Fig. 2. Before the treatment, the baseline of LVMI was the same between different groups (Fig. 2c). The HWI (Fig. 2b) and LVMI (Fig. 2d) were significantly greater in the DOX treated group than those in the control group due to the decrease of body weight. After the treatment for 2 weeks by SMY, HWI and LVMI were respectively recovered to 3.49 ± 0.145 mg/g and 2.67 ± 0.21 mg/g, with a statistical significance (*P* <0.05).

### Effects of SMY on physiological indexes

**Fig. 3:**
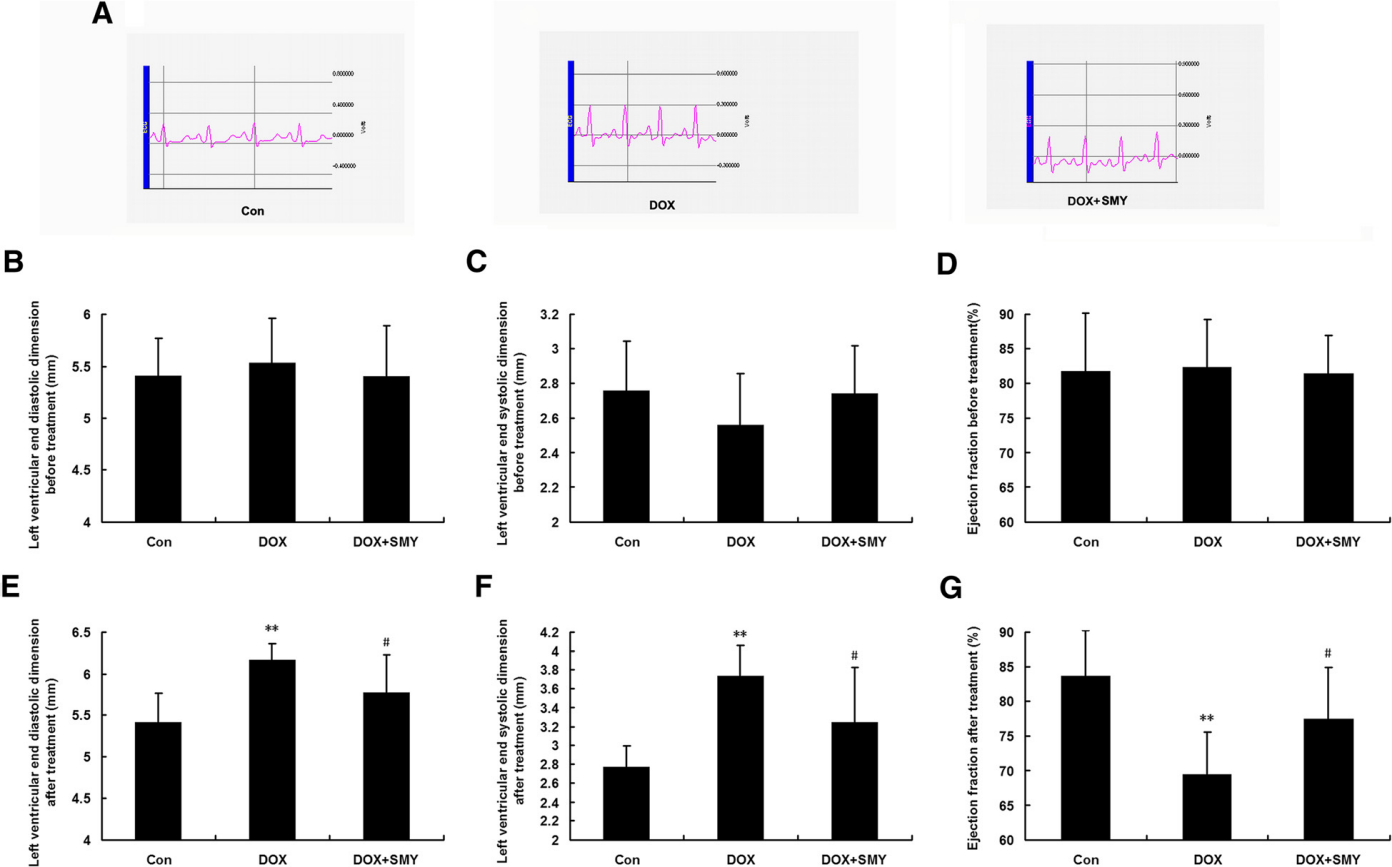
Electrocardiogram changes (**a**) in different groups. Baseline of left ventricular end diastolic dimension (**b**), left ventricular end systolic dimension (**c**) and ejection fraction (**d**) before the onset of the experiments. Effects of SMY on left ventricular end diastolic dimension (**e**), left ventricular end systolic dimension (**f**) and ejection fraction (**g**) in rats with DOX-induced cardiomyopathy. Ten rats in each group were analyzed in this experiment and all data were represented as mean ± standard deviation. ** *P* < 0.01 versus the group control; #*P* < 0.05 versus the group DOX

The results of our physiological studies were summarized in Fig. 3. The baselines of cardiac function in each group have no significant difference (Fig. 3b-d) before DOX or SMY treatment was administered. Rats receiving DOX alone had significant cardiac functional deterioration, characterized by increased heart rate, ST segment depression, elevated T wave (Fig. 3a); and signs of decreased cardiac function, i.e. increased LVEDD and LVESD, decreased EF as compared with control animals. Treatment with SMY significantly mitigated the DOX induced impairment of cardiac function, characterized by the reversal of LVEDD (Fig. 3e), LVESD (Fig. 3f) and EF (Fig. 3g).

### Effects of SMY on morphological changes and fibrosis related proteins

**Fig. 4:**
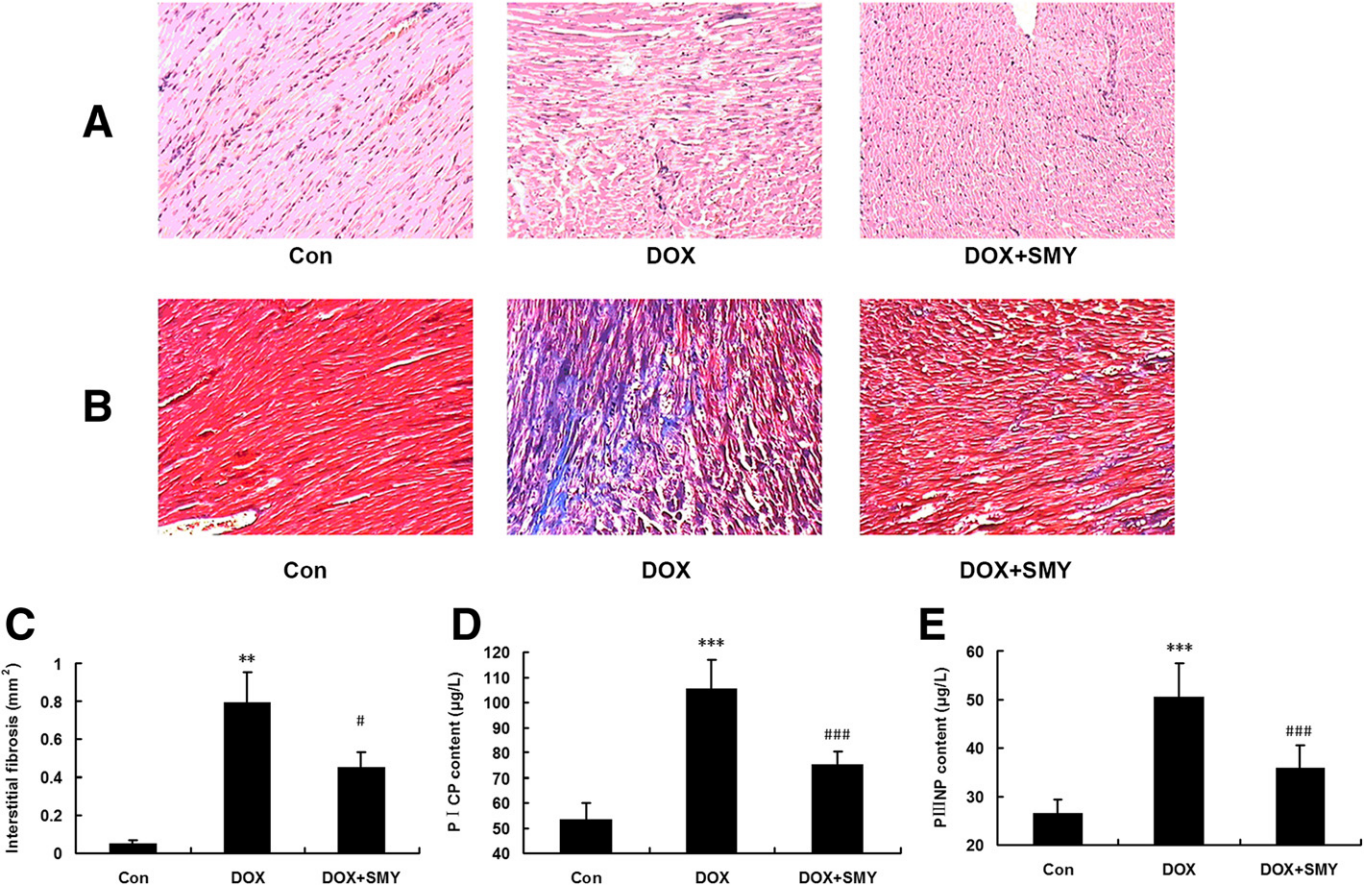
Representative photomicrographs of H&E staining on myocardial morphology (**a**) and interstitial fibrosis (**b**), with fibrotic tissue in blue and healthy cardiac tissue in pink and vascular fibrosis. Bar graph (**c**) showed a significant decrease in interstitial fibrosis (mm^2^) by SMY. Three rats in each group were analyzed in this experiment. (**d**) Effects of SMY on PICP, and PШNP (**e**) in rats with DOX-induced cardiomyopathy detected by ELISA. Six rats in each group were analyzed in ELISA assay and all data were represented as mean ± standard deviation. ** *P* < 0.01 versus the group control, *** *P* < 0.001 versus the group control; #*P* < 0.05 versus the group DOX, ###*P* < 0.001 versus the group DOX

Hhistopathological study (Fig. 4a) showed that rats in the control group had normal tissue morphology. As shown in DOX group rats, many myocardial structure disorders of muscle fibers and the infiltration of acute inflammatory cells were observed. The architecture in the rats treated by SMY was recovered to some extent. In addition, little amount of collagen was found in the interstitial and perivascular space in normal control rats (Fig. 4b). Under microscope, there was a large accumulation of collagen in the ventricle of DOX-injured rats, stained by blue. Less collagen deposition was found in SMY groups than that in DOX group. The bar graph in Fig. 4c showed a significant decrease in interstitial fibrosis (mm^2^) in the rats who received SMY treatment. In order to clarify the material basis of morphological changes further, we continued to explore the proteins correlated with myocardial fibrosis, such as PICP and PIIINP. DOX significantly increased the secretion of PICP (Fig. 4d: 1.98-fold, *P* < 0.001) and PШNP (Fig. 4e: 1.9-fold, *P* < 0.001). Treatment for 2 weeks by SMY resulted in suppression of PICP and PIIINP contents; with a reduction of 28.75 % and 29.05 % respectively.

### Effects of SMY on TGF-β1

**Fig. 5:**
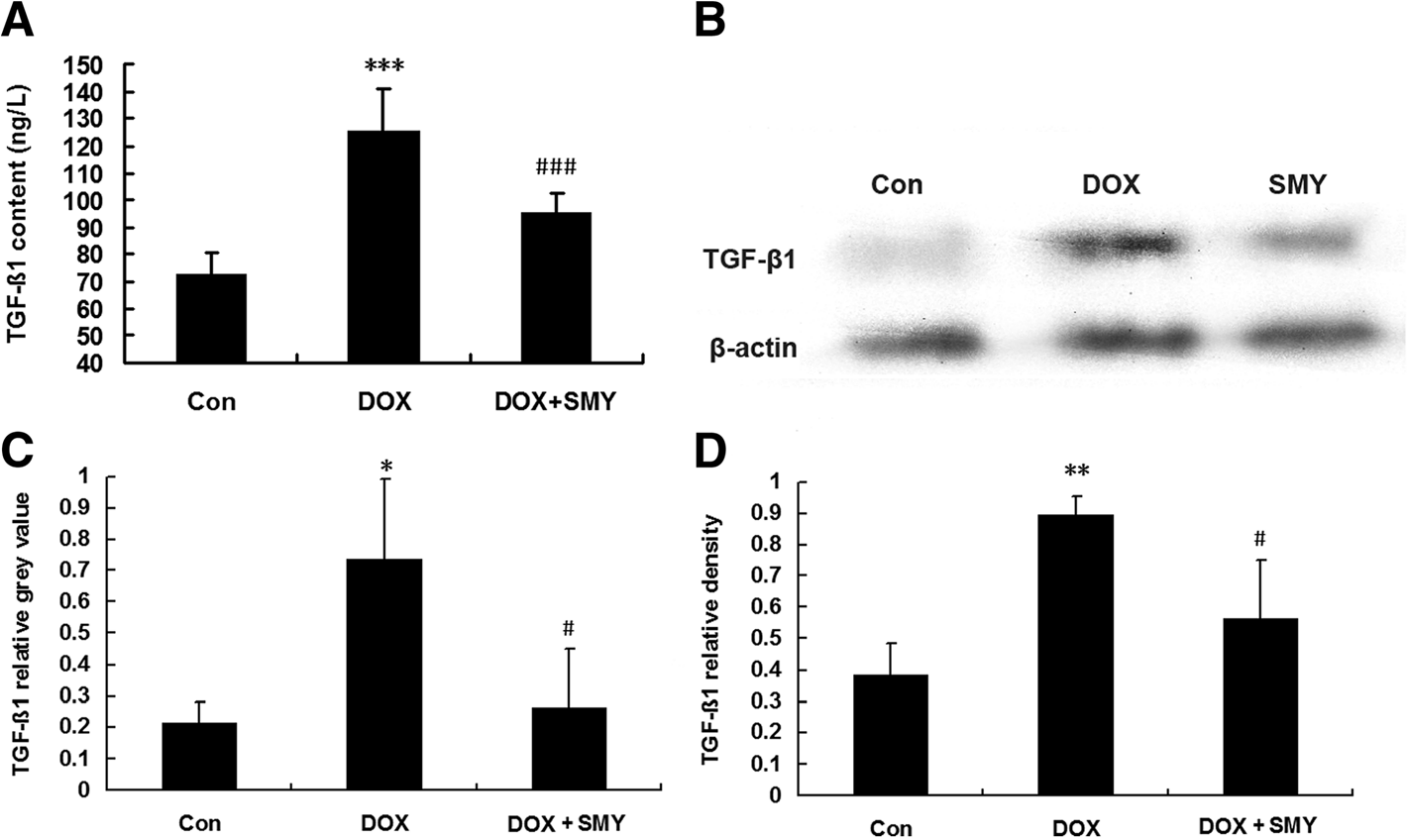
**a** Effects of SMY on TGF-β1 levels in rats with DOX-induced cardiomyopathy detected by ELISA which included six rats in each group. **b** Immunoblots of left ventricle were probed with anti- TGF-β1, while anti-β-actin antibody served as the loading controls. **c** Changes in TGF-β1 mRNA expression in left ventricle. **d** Bar graphs showed the relative density of TGF-β1 bands in left ventricle. Three rats were analyzed in PCR and western blots experiments. All data were represented as mean ± standard deviation. *** *P* < 0.001 versus the group control; ###*P* < 0.001 versus the group DOX

As shown in Fig. 5a, DOX significantly increased the level TGF-β1 in the serum detected by ELISA (1.73-fold, *P* < 0.001) which was significantly inhibited by SMY, with a reduction of 23.87 % (*P* < 0.001). The levels of TGF-β1 in both mRNA (Fig. 5c) and protein (Fig. 5b) were up-regulated in the DOX group by 3.4-fold (*P* < 0.05) and 2.34-fold (*P* < 0.05) respectively as compared with the control in response to DOX. Compared with the DOX group, SMY significantly down-regulate both mRNA level and protein level of TGF-β1 by 64.36 % and 37.1 %, respectively (*P* < 0.05). Data from immunostaining and quantification of TGF-β1 were also shown in Fig. 5d. The change of mRNA was consistent with that of protein which indicated that the regulation of SMY for the TGF-β1 was mediated by transcription mechanism.

### Effects of SMY on inflammatory factors

**Fig. 6:**
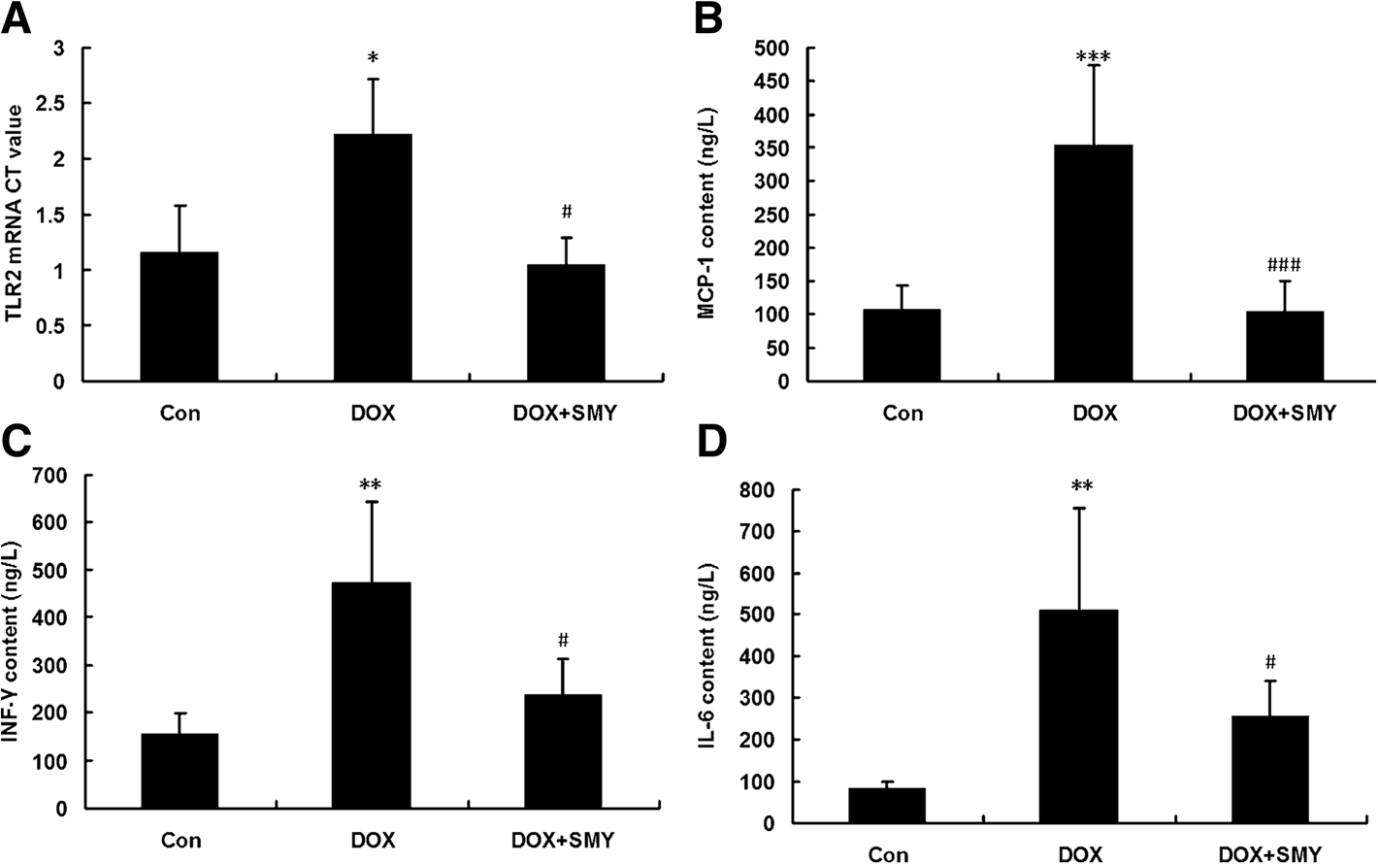
**a**. TLR2 mRNA level in DOX-injured cardiac tissue was reversed by SMY remarkably. Three rats were analyzed in PCR experiment. Meanwhile, the contents of MCP-1 (**b**), INF-γ (**c**) and IL-6 (**d**) were also inhibited significantly by SMY as compared with group DOX. Six rats were analyzed in ELISA assay and all data were represented as mean ± standard deviation. * *P* < 0.05 versus the group control, ** *P* < 0.01 versus the group control, *** *P* < 0.001 versus the group control; #*P* < 0.05 versus the group DOX, ###*P* < 0.001 versus the group DOX

Bar graphs in Fig. 6 displayed that DOX administration induced a high expression of TLR2 mRNA (Fig. 6a) and the amounts of inflammatory factors of MCP-1 (Fig. 6b), INF-γ (Fig. 6c) and IL-6 (Fig. 6d) as compared with the control group (*P* < 0.05, *P* < 0.01 or *P* < 0.001). When exposed under SMY, the TLR2 mRNA level was down-regulated by 47.11 %. The contents of MCP-1, INF-γ and IL-6 in SMY-treated heart tissue were also suppressed significantly as compared with DOX group, with reduction of 70.48 %, 50.35 % and 50.17 %, respectively (*P* < 0.05 or *P* < 0.001).

## Discussion

In spite of being an effective anti-cancer agent, DOX usage at maximal therapeutic dose is life threatening due to its accumulation in the circulation which may induce irreversible cardiomyopathy and heart failure [22]. Following DOX treatments, aberrations to myocardial architecture and function include cardiac cell hypertrophy and death, heightened susceptibility to myocardial infarction (MI), cardiomyopathy, and left ventricular dysfunction [23–25]. Many CAMs show significant improvements in chemotherapy- or radiotherapy-related side effects and TCM is the most common means of CAM in China. Shengmai, as a traditional Chinese medicine, is usually used in China as a complementary treatment to western treatments that are recommended for acute heart failure, and also as a single treatment for chronic heart failure [26]. Previous studies have focused on the study of SMY on ischemic cardiomyopathy [27, 28] or contractile function of aged hearts [17]. There are few reported studies about SMY on non-ischemic cardiomyopathy, such as DOX-induced cardiomyopathy and heart failure. Our study provides the important evidence of the beneficial effects of SMY on cardiac dysfunction resulting from DOX-induced heart injury in terms of myocardial fibrosis. We showed a dose-dependent cardioprotecion of SMY in a rat model induced by DOX, characterized by the suppression of BNP and CK-MB, the biomarkers for myocardial infarction and the biochemical criteria of myocardial infarction diagnosis. In agreement with the outcomes of previous studies, DOX induced signs of cardiomyopathy in the form of decreased BW, increased heart index and left ventricular mass index, indicating a severe dysfunction in cardiac performance [29, 30]. We found that administration of clinical equivalent dose of SMY during the injury of DOX significantly increased body weight, decreased the HWI and LVMI which suggested that this formula was an effective and safe strategy for cardiotoxicity.

Echocardiography is a versatile noninvasive tool for measuring cardiac function and structure in animal as well as in clinic [31, 32]. The change of T-wave in the ECG is an important signal that reflects the extent of heart injury [33, 34]. In our study, the T-wave of the rats was elevated after DOX administration. We used echocardiography to estimate LV function as reflected by LVEDD, LVESD and EF. The echocardiography records showed that the baseline of LV function before treatment was consistent, with no statistically significant difference. SMY significantly attenuated the change of T-wave amplitude. In addition, SMY also decreased the LVEDD and LVESD, as well as increased EF, suggesting that SMY can prevent DOX-induced heart function deterioration.

Fibrosis plays a major role in adverse cardiac remodeling in DOX-induced cardiomyopathy (DIC) and post-MI myocardium [35]. The ultra-structural changes seen in endomyocardial biopsies of patients with DOX-associated heart failure include loss of myofibrils, disarray of sarcomere structure, dilation of the sarcoplasmic reticulum, swelling of the mitochondria and cytoplasmic vacuolization [36]. As expected, the degree of cardiac injury was displayed by myocardial structure disorders of muscle fibers as well as infiltration of acute inflammatory cells, elevated quantities of interstitial and vascular fibrosis, demonstrable microscopically with an up to approximately 15.8-fold increase in interstitial fibrosis. Our data further suggested that following SMY administration, accumulation of collagen was attenuated. This may be one of the main reasons of the improvement of LVEDD, LVESD and EF.

Serum levels of PICP and PIIINP are the markers of myocardial fibrosis. Type I collagen is a major component of myocardial extracellular matrix and plays a key role in ventricular remodeling [37]. The other type of collagen associated with ventricular remodeling is collagen type III (PIIINP), a serum biomarker of myocardial biosynthesis of type III collagen is associated with poor left ventricular function and high mortality following myocardial infarction [38]. Fibrosis plays a major role in adverse cardiac remodeling in DIC and post myocardial infarction [35]. In our paper, we showed that the markers of myocardial fibrosis (PICP and PIIINP) were increased after the DOX. The serum PICP was depressed in rats administrated with SMY, suggesting the collagen turnover may have dropped to a lower level. Although the value of serum PIIINP in predicting the DIC is uncertain, the level of PIIINP is still used as indicators of myocardial fibrosis in vivo to clarify the influence on myocardial fibrosis [39]. The administration of SMY for 2 weeks during DOX injury significantly decreased the content of PIIINP in serum which indicated that SMY attenuated myocardial fibrosis to some extent.

TGF-β1 is a protein secreted by cardiac myofibroblast and fibroblast that controls proliferation and is responsible for cardiac apoptosis, hypertrophy, and fibrosis [40]. Previous report showed that TGF-β1 gene expression is increased in the left ventricular myocardium of patients with idiopathic hypertrophic cardiomyopathy or dilated cardiomyopathy and in animals after myocardial infarction [41]. In the present study, treating rats with DOX not only expressed high content of TGF-β1 in serum, but also exhibited high expression in myocardium. These findings suggest the possible involvement of TGF-β1 gene and protein in the regulation of DOX-cardiotoxicity process. We further explored the mRNA and protein levels of TGF-β1 treated by SMY and found that the down-regulation of TGF-β1 mRNA by SMY was consistent with the protein levels which indicated that the regulation of SMY for TGF-β1 was mediated through transcriptional mechanism. Combined with the other results in this paper, we can speculate that the improvement of cardiac function and morphology by SMY maybe mediated by the suppression of fibrosis associated proteins and gene.

The occurrence and development of chronic inflammation in chronic heart failure is considered to be the initiating factor in heart tissue fibrosis [42]. The inflammatory response caused by injury initiated the processes of tissue repair which could cause tissue fibrosis. Blockade of TLR2 attenuated LV dysfunction and fibrosis in DOX triggered acute and chronic cardiomyopathy, which was strongly associated with the reduced inflammation and TLR2 endogenous agonist levels [43]. Stimulation of TLR2 induces a mixed Th1, Th2, Treg, and Th17 types of immune response, which mainly mediates the chronic inflammatory and tissue injury responses [44]. To clarify the inflammation mechanism, we detected TLR2 mRNA and concerned inflammatory factors. Treatment of DOX-injured rats with SMY reduced TLR2 mRNA expression and the levels of MCP-1, IL-6 (a typical Th2-type cytokine) and INF-γ (a typical Th1-type cytokine). Combined with TGF-β (a Treg cytokine), our analysis for the Th1/Th2/Treg suggested that anti-inflammation and the regulation for cardiac immune microenvironment might be other possible mechanisms for the reversal of myocardial fibrosis by SMY.

## Conclusions

SMY exhibited cardioprotection on DOX in rats, which might be attributable to the improvement of heart function, the attenuation of myocardial fibrosis and the down-regulation of fibrosis associated protein. Further studies are required to identify the mechanisms behind the SMY.
